# Comprehensive microRNA profiling in acetaminophen toxicity identifies novel circulating biomarkers for human liver and kidney injury

**DOI:** 10.1038/srep15501

**Published:** 2015-10-22

**Authors:** A. D. B. Vliegenthart, J. M. Shaffer, J. I. Clarke, L. E. J. Peeters, A. Caporali, D. N. Bateman, D. M. Wood, P. I. Dargan, D. G. Craig, J. K. Moore, A. I. Thompson, N. C. Henderson, D. J. Webb, J. Sharkey, D. J. Antoine, B. K. Park, M. A. Bailey, E. Lader, K. J. Simpson, J. W. Dear

**Affiliations:** 1 Pharmacology, Toxicology and Therapeutics, University/BHF Centre for Cardiovascular Science, Edinburgh University, UK; 2 Qiagen, Fredrick, Maryland, USA; 3 MRC Centre for Drug Safety Science, Department of Molecular & Clinical Pharmacology, Institute of Translational Medicine, University of Liverpool, Liverpool, UK; 4 Clinical Toxicology, Guy’s and St Thomas’ NHS Foundation Trust, London, UK; 5 King’s College London, London, UK; 6 Scottish Liver Transplantation Unit, Royal Infirmary of Edinburgh, Edinburgh, UK; 7 MRC Centre for Inflammation Research, University of Edinburgh, The Queen's Medical Research Institute, 47 Little France Crescent, Edinburgh

## Abstract

Our objective was to identify microRNA (miRNA) biomarkers of drug-induced liver and kidney injury by profiling the circulating miRNome in patients with acetaminophen overdose. Plasma miRNAs were quantified in age- and sex-matched overdose patients with (N = 27) and without (N = 27) organ injury (APAP-TOX and APAP-no TOX, respectively). Classifier miRNAs were tested in a separate cohort (N = 81). miRNA specificity was determined in non-acetaminophen liver injury and murine models. Sensitivity was tested by stratification of patients at hospital presentation (N = 67). From 1809 miRNAs, 75 were 3-fold or more increased and 46 were 3-fold or more decreased with APAP-TOX. A 16 miRNA classifier model accurately diagnosed APAP-TOX in the test cohort. In humans, the miRNAs with the largest increase (miR-122-5p, miR-885-5p, miR-151a-3p) and the highest rank in the classifier model (miR-382-5p) accurately reported non-acetaminophen liver injury and were unaffected by kidney injury. miR-122-5p was more sensitive than ALT for reporting liver injury at hospital presentation, especially combined with miR-483-3p. A miRNA panel was associated with human kidney dysfunction. In mice, miR-122-5p, miR-151a-3p and miR-382-5p specifically reported APAP toxicity - being unaffected by drug-induced kidney injury. Profiling of acetaminophen toxicity identified multiple miRNAs that report acute liver injury and potential biomarkers of drug-induced kidney injury.

Acetaminophen (paracetamol) is a safe analgesic drug when taken at therapeutic doses. However, in overdose acetaminophen is hepatotoxic and is the most common cause of acute liver failure in the United States and Europe^1^,^2^. After overdose, the reactive metabolite N-acetyl-p-benzoquinone imine (NAPQI) is generated in excess, depleting cellular glutathione (GSH) then binding covalently to cellular proteins resulting in oxidative stress and hepatocyte death, predominately by necrosis^3^. Cell death releases intra-cellular molecules into the extra-cellular milieu and this is reflected by changes in circulating protein and RNA^4^. The current antidote, acetylcysteine (NAC), replenishes cellular GSH and is highly effective at preventing liver injury if administered soon after overdose^5^. However, NAC treatment takes at least 21 hours to complete so resulting in substantial hospital bed occupancy and commonly producing adverse drug reactions; 65% of NAC-treated patients vomited, retched or needed antiemetic therapy in a recent randomised controlled trial^6^. To selectively target treatment to those who stand to benefit and facilitate early safe hospital discharge of low risk patients there is an unmet need for new biomarkers that stratify patients by their risk of liver injury at first presentation to hospital, soon after overdose, an early time point when current markers such as serum alanine transaminase (ALT) activity lack sensitivity and specificity^7^.

Acetaminophen can also induce kidney tubular cell death resulting in acute kidney injury (AKI)^8^ and, in the presence of liver injury, AKI is one of the key predictors of need for urgent liver transplantation to avoid death. Kidney injury is currently quantified by serum creatinine. However, patients with AKI are not in steady-state with regard to kidney function and serum creatinine is slow to report cellular damage. Serum creatinine also lacks specificity, becoming elevated by non-renal pathologies such as dehydration and muscle injury^9^. New biomarkers are needed to report drug-induced kidney injury with enhanced sensitivity and specificity.

MicroRNAs (miRNAs) are small (~22 nucleotide-long) non-protein coding RNA species involved in post-transcriptional gene-product regulation^10^. In blood, miRNAs are stable because they are protected from degradation by extra-cellular vesicles (such as exosomes), RNA binding protein complexes (such as argonaute 2 – Ago2) and high-density lipoproteins^11^,^12^. The different circulating carriers for miRNA may reflect different pathways of release from cells; exosomal miRNAs may represent physiological release whereas miRNAs bound to Ago2 are increased with cell necrosis. As they are amplifiable and some are tissue restricted, miRNAs represent a reservoir for biomarker discovery. Liver-enriched miR-122-5p is released by injured hepatocytes and is a circulating biomarker for liver toxicity in zebrafish^13^, rodents^14^ and humans^15^. In patients with acetaminophen-induced liver injury circulating miR-122-5p has been reported to be increased around 100-fold compared to controls^15^,^16^. However, it is not known whether other miRNAs can out-perform miR-122-5p with regard to patient stratification.

The field of profiling multiple circulating miRNAs to discover signatures of toxicity is relatively new. In rodent models of acetaminophen toxicity there have been profiling studies of relatively small numbers of circulating miRNAs - these studies demonstrate that multiple miRNA species change with liver injury^17^,^18^. In humans, a miRNome subset of 372 miRNAs was quantified in 49 patients with acetaminophen hepatotoxicity or ischemic hepatitis^17^. This study confirmed that miRNAs were increased and decreased and suggested that certain species could distinguish between these distinct aetiologies of liver injury^17^. Changes in circulating miRNAs have also been reported in small numbers of hepatotoxicity patients analysed by high-throughput sequencing^18^,^19^. These studies did not address the unmet clinical need for improved patient stratification, back translate to pre-clinical models or identify signals of kidney toxicity. In the present study we recruited over 200 patients and assayed miRBase version 18 to identify which miRNAs were differentially expressed in plasma from overdose patients with and without acetaminophen toxicity (APAP-TOX and APAP-no TOX, respectively). Selected miRNA candidate biomarkers were tested for liver and kidney specificity in humans and mice, and sensitivity with regard to patient stratification at first presentation to hospital.

## Results

### Circulating miRNAs that were differentially expressed with acute liver injury

**Figure 1:**
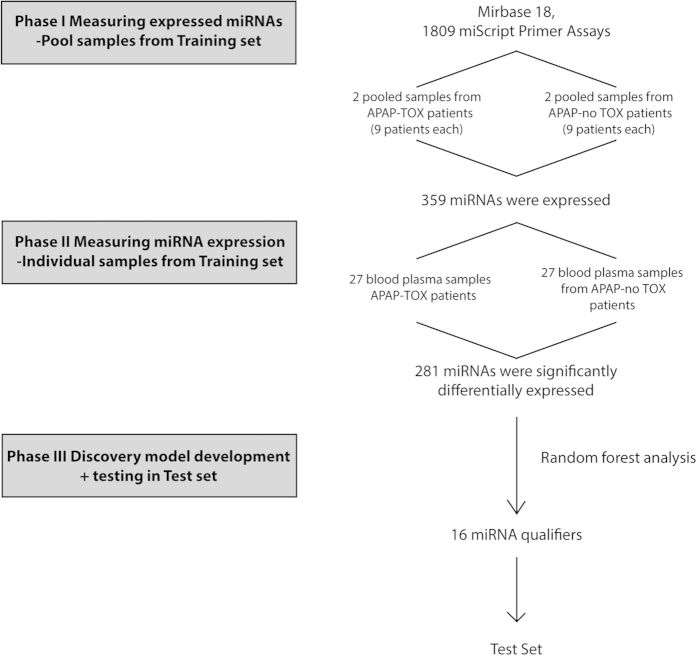
Study design. Phase I identified the expressed microRNAs (miRNAs) in pooled samples from acetaminophen toxicity (APAP-TOX) patients and acetaminophen overdose with no toxicity (APAP-no TOX) patients. In Phase II, expressed miRNAs were quantified in 27 APAP-TOX and 27 APAP-no TOX patients. For data processing, C_t_ values were calibrated for RNA recovery using the cel-miR-39-3p assay (which detects a synthetic miRNA spiked in to each sample during sample prep). In Phase III, random forest analysis was performed to develop the classifier model that was subsequently tested in the test set.

**Figure 2:**
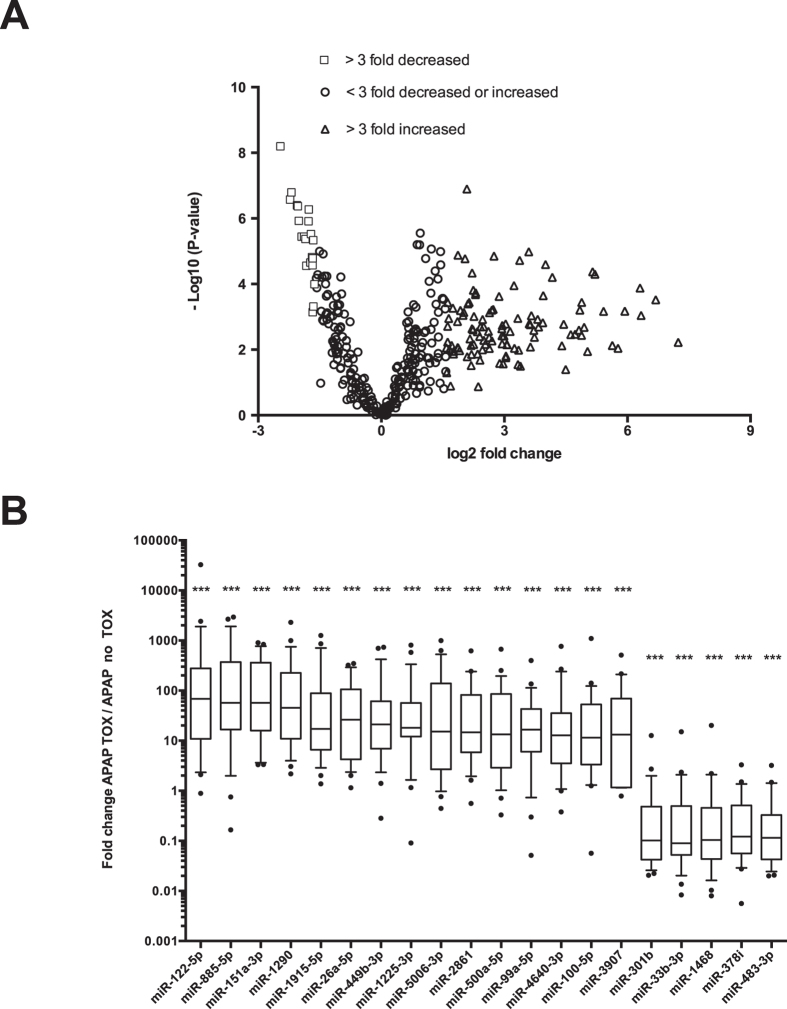
Differentially expressed circulating microRNAs (miRNAs) with toxicity. (**A**) Volcano plot of miRNA data when acetaminophen toxicity (APAP-TOX) patients were compared with age and sex matched acetaminophen no toxicity (APAP-no TOX) patients. The negative logarithm base 10 of the P-value is plotted against log 2 difference in estimated relative expression values. Respectively, the triangles and squares correspond to miRNAs that were more than 3 fold increased or decreased in the APAP-TOX patients relative to the APAP-no TOX patients. (**B**) Box-and-whisker plots displaying the top 15 highest fold increase miRNAs and 5 highest fold decrease miRNAs. The fold regulation was calculated using the 2^−ΔΔCt^ method. Statistically significant differences were determined by Wilcoxon matched-pairs signed rank test using 2^−ΔCt^. Ct values were normalized using global Ct mean. Statistically significant differences were determined by Mann Whitney Test using ΔCt values. ***P < 0.001; **P < 0.01; *P < 0.05.

**Figure 3:**
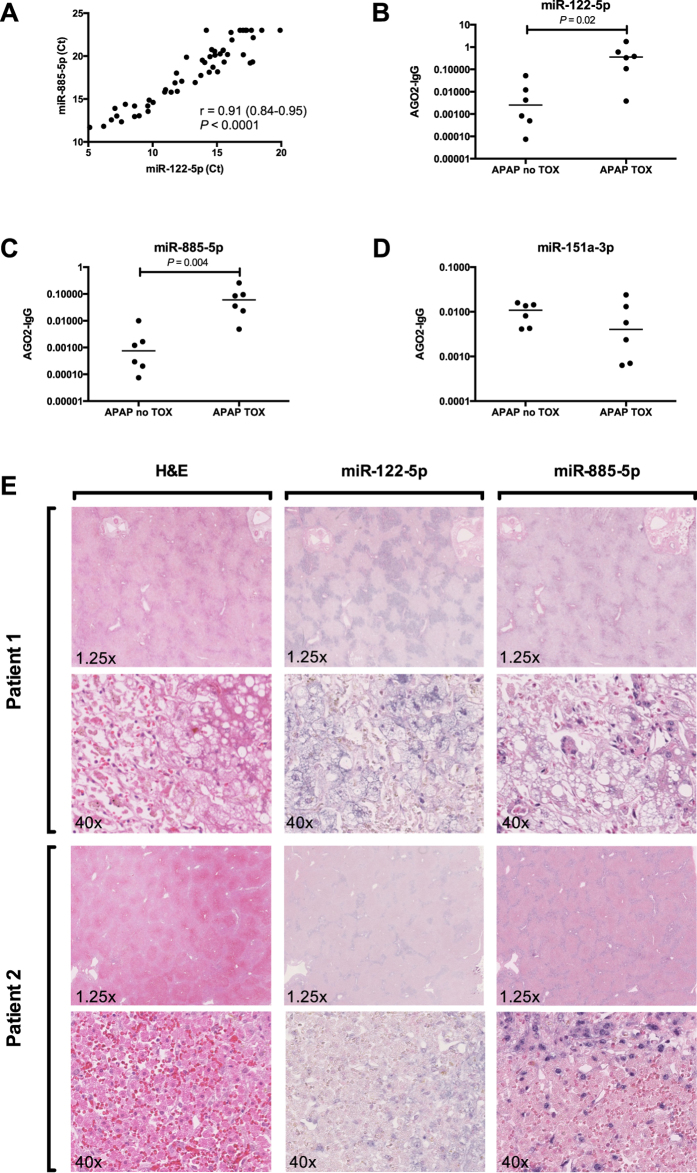
miR-122-5p and miR-885-5p are released from human hepatocytes bound to the carrier protein Ago2. Figure (**A**) Pearson correlation plot of circulating miR-885-5p and miR-122-5p across APAP-TOX and APAP-no TOX patients. Figure (**B**–**D**) represent the relative Ago2 fraction for miR-122-5p, miR-885-5p and miR-151a-5p respectively in APAP-TOX (N = 6) and APAP-no TOX (N = 6) patients. The Y axis represents the 2^−ΔCt^ value obtained from the Ago2 pull-down minus the 2^−ΔCt^ value obtained from the IgG pull-down from the same sample, both normalised by spiked-in synthetic miR-39. Statistically significant differences were determined by Mann Whitney Test. Figure (**E**) presents liver tissue from 2 patients whom underwent liver transplantation for severe acute liver failure due to acetaminophen overdose. Images from hematoxylin and eosin (H&E) and *in situ* hybridization for miR-122-5p and miR-885-5p are presented.

The experimental design used to identify differentially expressed miRNAs is presented in Fig. 1. In phase 1 of this discovery study, randomly selected APAP-TOX and APAP-no TOX samples were pooled and expressed miRNAs were identified. From 1809 miRNA species, 359 miRNAs fulfilled our criteria of expression (Ct value < 35 with a single, sharp melt peak) in at least one of the 4 sample pools (2 x APAP-TOX and 2 x APAP-no TOX). The patient demographics and clinical chemistry results from APAP-TOX and APAP-no TOX patients in the training set and test set are presented in supplementary Table 1. In Phase II, these 359 miRNAs were quantified in each of the 54 patient samples of the training set using a bespoke PCR array. The PCR results from phase II for each microRNA are presented in supplementary file 1. The PCR results from 1 sample in the APAP-TOX group did not have any amplification curves and this sample was excluded from further analyses. The phase II PCR profiling results: 75 miRNAs were 3-fold or more increased in the APAP-TOX patient group compared to the age- and sex-matched APAP-no TOX group. 46 miRNAs were 3-fold or more decreased in the APAP-TOX patient group. These data are presented in Fig. 2A as a volcano plot. The largest median (IQR) increased circulating miRNAs were miR-122-5p 68 (11–277), miR-885-5p 57 (17–372) and miR-151a-3p 57 (16–360) (Fig. 2B). A heatmap with cluster analysis of >3-fold increased/decreased circulating miRNAs in the APAP-no TOX and APAP-TOX patients from the training set is displayed in supplementary Figure 1. This heatmap demonstrates that APAP-TOX patients can be clustered into sub-groups based on the expression of miRNA panels. There was a group of patients with lower miR-30b-5p, miR-186-5p, miR-382-5p, miR-27a-3p, miR-15a-3p and miR15a-5p. These APAP-TOX patients were more unwell (INR 2.0 (16-3.4) v 1.4 (1.2–1.5) in rest of APAP-Tox group P = 0.003; serum creatinine 152 mg/dl (64–289) v 61 mg/dl (58–67) P = 0.01). The largest fold increase miRNAs (miR-122-5p and miR-885-5p) were highly correlated across patients in the training set (Fig. 3A). miR-122-5p has been reported to circulate in a protein fraction of plasma rather than in extra-cellular vesicles although this has not been characterized in humans. After antibody-mediated pull down of Ago2 (corrected by IgG control), acetaminophen toxicity induced a significant increase in the amount of miR-122-5p and miR-885-5p specifically bound to Ago2 (Fig. 3B,C), consistent with both miRNAs being released bound to this protein. Interestingly there was no increase in miR-151a-3p, which suggests different mechanisms of release across miRNA species (Fig. 3D). *In situ* hybridization for miR-122-5p and miR-885-5p was performed on liver explants removed following acetaminophen overdose. Both these miRNAs localised to hepatocytes and their expression was reduced in the areas of centrilobular necrosis, consistent with release from injured cells (Fig. 3E).

### Development and testing of miRNA diagnostic panels

**Figure 4:**
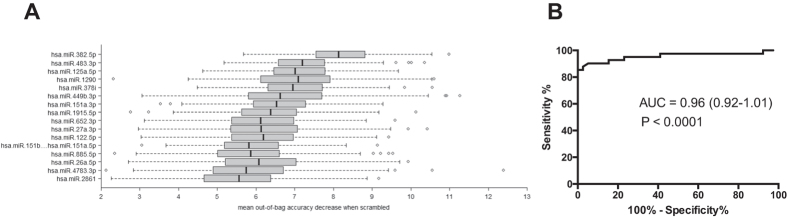
Random forest statistics analysis. Figure (**A**) presents the top 16 miRNAs that separate APAP-TOX from APAP-no TOX in the training set. miRNAs were ranked according to the marginal decrease in out-of-bag prediction accuracy when the gene’s expression measurements are scrambled. Figure (**B**) Receiver operator characteristic curve displaying the performance of the top 16 miRNAs from the training set with regard to distinguishing APAP-TOX from APAP- no TOX in the test set.

In Phase III random forest statistics were used to identify which miRNAs separate APAP-TOX from APAP-no TOX. The analysis demonstrated that 16 miRNAs (‘classifier model’) had the lowest prediction error - the largest marginal decrease in prediction accuracy when their values are randomly permuted (Fig. 4A). This classifier model was tested in an independent test set of 81 patient samples (Fig. 4B). The probability of a sample being correctly classified by the miRNA model as APAP-TOX was significantly higher when the true classification was APAP-TOX (median percent probability (IQR): 75 (63–77) for true APAP-TOX; 44 (43–47) for true APAP-no TOX; P < 0.0001). For an APAP-TOX classification probability cut-off of 50% (i.e. higher probability of APAP-TOX than APAP-no TOX) the classifier series sensitivity was 90% (95% CI: 76 to 97) and specificity was 90% (77 to 97) (Fig. 4B).

### Specificity of candidate miRNA biomarkers

The 3 largest fold increase miRNAs (miR-122-5p, miR-885-5p and miR-151a-3p) and the miRNA with the lowest prediction error from the classifier model (miR-382-5p) were taken forward and tested for specificity and sensitivity.

#### Human specificity studies

**Figure 5:**
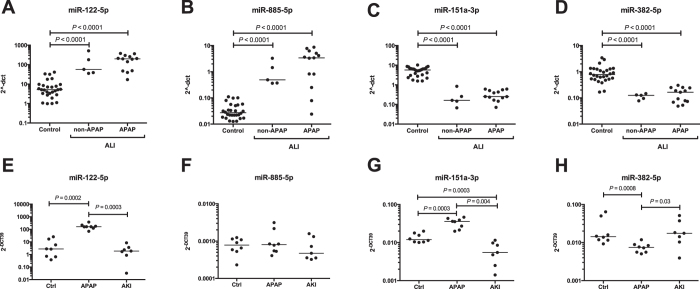
Biomarker specificity for liver injury. Figure (**A**–**D**) present circulating miR-122-5p, miR-885-5p, miR-151a-3p and miR-382-5p in APAP-no TOX patients and patients with acute liver injury (ALI) induced by APAP overdose or another aetiology (non-APAP). Figure (**E**–**H**) present miR-122-5p, miR-885-5p, miR-151a-3p and miR-382-5p in control mice, APAP overdose mice and cisplatin-induced acute kidney injury (AKI) mice. Values represent 2^−ΔCt^ normalised by miR-1287 in human samples and spiked in synthetic miR-39 in mice samples. Statistically significant differences were determined by Wilcoxon matched-pairs signed rank test.

In patients with acute liver injury (ALI) due to a diverse range of non-acetaminophen causes, all 4 miRNA species were significantly changed in their circulating concentration to levels comparable with the APAP-TOX group (Fig. 5A–D). The kidney is commonly injured in the setting of ALI so could be the tissue of origin for circulating miRNAs and kidney dysfunction could affect the circulating concentration of liver-derived miRNAs by altering their clearance. Two complementary approaches were used to determine miRNA liver specificity in the presence of kidney injury. APAP-TOX patients without and with kidney dysfunction (serum creatinine concentration >110 μmol/L) were compared across the training and test sets (supplementary Table 2). There was an increase in the median PCR-array Ct value with kidney dysfunction, consistent with a overall reduction in circulating miRNAs (APAP-TOX no AKI: 16.55 (15.78–17.49); APAP-TOX AKI: 18.01 (16.84–19.02). P = 0.0007). However, there was no difference in miR-122-5p, miR-885-5p, miR-151a-3p or miR-382-5p (Table 1). The miRNAs that were significantly changed with kidney dysfunction are presented in Table 1.

#### Mouse specificity studies

Mouse models of acetaminophen and cisplatin toxicity were compared with regard to miRNA circulating concentrations, cisplatin toxicity being a well-established model of drug-induced AKI. Acetaminophen induced liver injury was confirmed by a median (IQR) serum ALT activity of 7230IU/L (5355–23615, N = 8) and histologically by centrilobular hepatic necrosis. By contrast with vehicle treated controls (N = 7), acetaminophen toxicity in mice resulted in increased miR-122-5p and miR-151a-3p, and decreased miR-382-5p, in line with our human data (Fig. 5E–H). However, miR-885-5p was not translatable across species and did not change. Cisplatin-induced AKI was demonstrated by significant elevations in urinary KIM-1 concentration (control: 1322 pg/mgUCr (766–1460); cisplatin 10,964 pg/mgUCr (4493–16003). N = 7, P = 0.0003) and histologically, there were apoptotic proximal tubular cells, sloughing and an overall reduced number of epithelial cells. Cisplatin had no effect on miR-122-5p, miR-885-5p, miR-151a-3p or miR-382-5p (Fig. 5E–H).

#### Human case report

**Figure 6:**
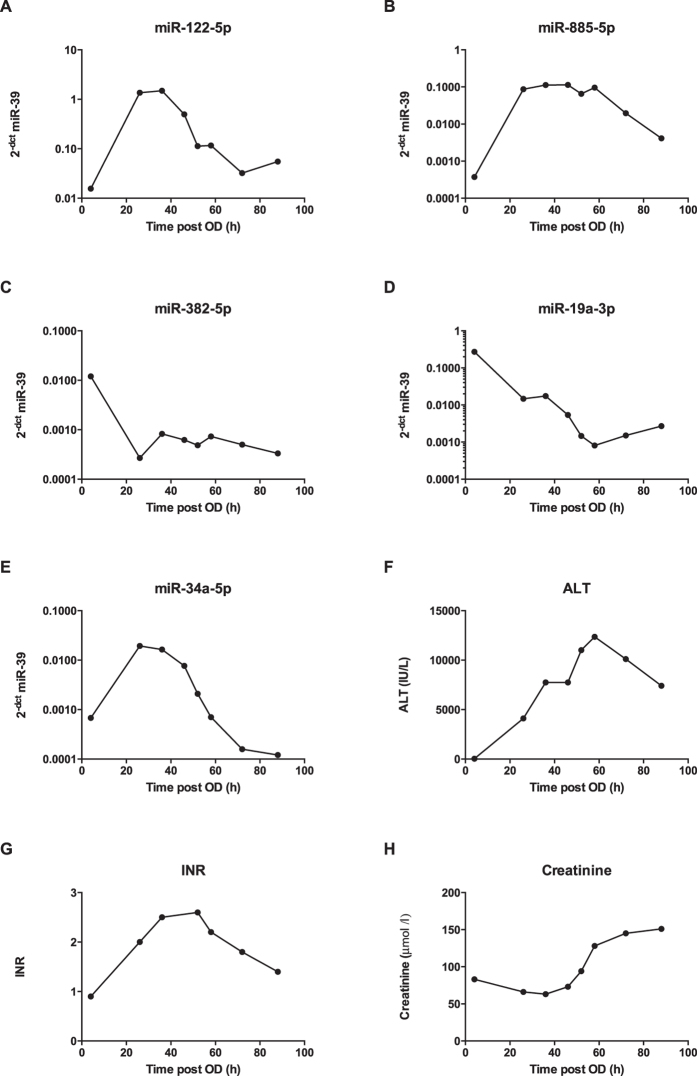
Timeline of current and novel miRNA biomarkers following a large acetaminophen overdose in a 49 year-old male. Values for miRNA represent 2^−ΔCt^ normalised by spiked-in synthetic miR-39.

To further define temporal changes, miRNA candidate biomarkers were measured in serial samples from a 49 year-old man who presented to hospital soon after a large overdose (4 hour acetaminophen concentration: 405 mg/L) with initially normal serum ALT activity (28 U/L) and kidney function (serum creatinine: 83 μmol/L). Despite prompt NAC treatment (started 6 hours after overdose), the patient subsequently developed significant liver injury (peak ALT: 12,353 U/L) with coagulopathy (peak INR: 2.8) and kidney injury (peak creatinine: 151 μmol/L) (Fig. 6F–H). The patient’s liver injury recovered with continued NAC treatment, providing an opportunity to track miRNA concentrations from no apparent injury, into significant organ injury and through to liver recovery. The temporal profiles of the individual miRNAs were different; miR-122-5p concentration was elevated but decreased earlier than ALT (Fig. 6A). miR-885-5p remained elevated longer than miR-122-5p (Fig. 6B). In line with the array data, both miR-382-5p and miR-19a substantially decreased in concentration and, interestingly, they remained below the hospital admission level (Fig. 6C,D). miR-34a initially increased but then fell to below the admission concentration, a decrease which corresponded temporally with increased serum creatinine concentration (Fig. 6E).

### Identification of endogenous miRNA normalizers

To identify which miRNAs were most stably expressed across APAP-TOX and APAP-no TOX patients NormFinder analysis was performed on the training set data. miR-1287 was the most stably expressed with a stability value of 1.22*10−5. All results from the NormFinder assessment are reported in supplementary Table 3. miR-1913, miR-671, miR-1287, Let-7d, miR-1260 and miR-324 were taken forward and measured in the APAP-early patient samples using RT-PCR. The demographics and standard clinical chemistry for this patient cohort are presented in supplementary Table 4. In accordance with the NormFinder assessment, miR-1287 was most stably expressed across the different samples with a coefficient of variation (CV) of 1.71% (supplementary Figure 2).

### Sensitivity - comparison of leading microRNA discriminators with ALT as a diagnostic of early ALI

**Figure 7:**
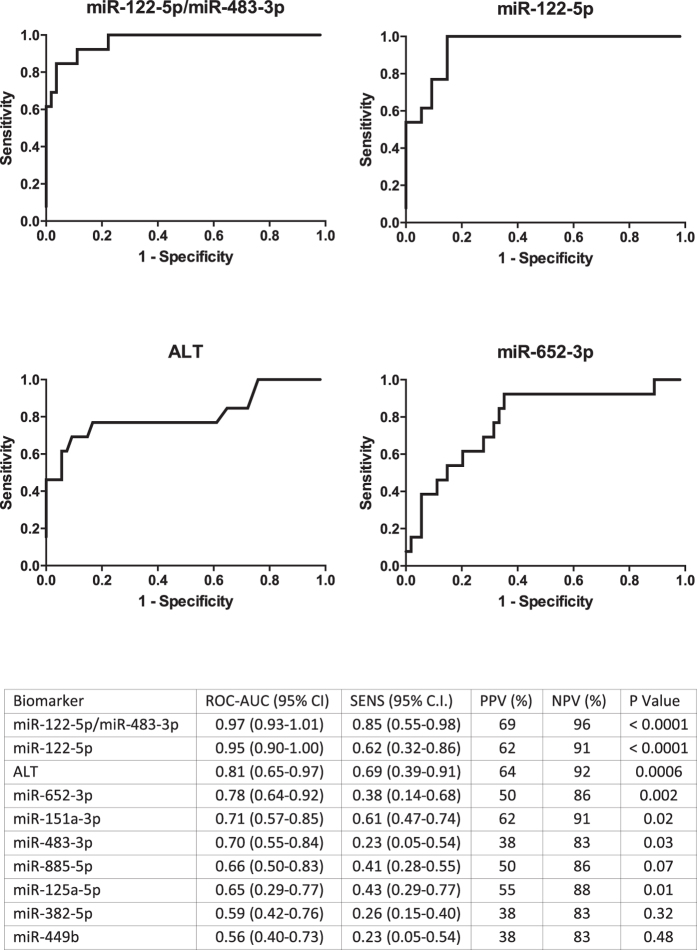
Biomarker sensitivity - ROC curve analysis supports the potential for miR-122-5p to predict the development of ALI. ROC analysis was calculated to determine the potential for plasma miRNAs and serum ALT activity to predict the development of TOX at first presentation to hospital following acetaminophen overdose. TOX was defined as >3xULN ALT activity during hospital admission. The ROC curves of the 4 most accurate predictors are presented. AUC (area under the curve with 95% CI), sensitivity (SENS at 90% specificity) with 95% CI, positive and negative predictive values (PPV and NPV) and statistical significance for AUC value is presented in table. The miRNAs are normalized by miR-1287.

In the 67 APAP-early patients, miR-122-5p identified subsequent liver injury when normalized by any of the 6 endogenous miRNA normalizers described above (miR-122-5p area under ROC curve normalized by miR-1913, 0.97 (95% CI 0.92–1.01); miR-671, 0.96 (0.92–1.01); miR-1287, 0.95 (0.90–1.00); let7-d, 0.94 (0.89–1.00); miR-1260, 0.93 (0.88–1.00); miR-324, 0.93 (0.87–1.00) miR-122-5p ROC-AUC significantly larger than all other miRNAs – P < 0.05). miR-885-5p, miR-151a-5p and miR-382-5p were inferior to ALT for early prediction of liver injury (Fig. 7). When the largest fold increase and decrease phase II miRNAs were combined (miR-122-5p and miR-483-3p, respectively) there was a modest increase in the area under the ROC curve compared with miR-122-5p alone. No other miRNA combination improved predictive accuracy (data not shown).

## Discussion

To date, this is the largest and most comprehensive study of the circulating miRNome in humans with acetaminophen toxicity. We identified multiple miRNA species that separated APAP-TOX from APAP-no TOX in a training set of patients, then tested the panels in a separate patient cohort. The large number of miRNAs that were screened in this study (1809) allowed the identification of novel miRNA species that report toxicity. The largest fold change miRNAs (miR-122-5p, miR-885-5p and miR-151-3p) and the best discriminating miRNA (miR-382-5p) were taken forward and tested for specificity. Although variable, they report acute liver injury across patients and mouse models, even in the presence of kidney injury. Kidney injury resulted in significantly decreased miRNAs that represent new circulating biomarker candidates. The most stable internal miRNA normalizers were discovered and used to normalize miRNAs to compare with ALT activity with regard to sensitivity (the stratification of patients at first presentation to hospital). miR-122-5p very accurately predicted liver injury at first presentation to hospital, especially when combined with the largest decrease miRNA.

This study reported that 75 circulating miRNA species were more than 3-fold increased with acute liver injury, demonstrating the potential of multiple miRNA species as biomarkers. miRNAs identified in the training set of patients were validated in a separate patient cohort from a separate clinical unit. This 16-miRNA ‘signature’ of acute liver injury accurately identified toxicity. The most abundant miRNA species in the liver^20^, miR-122-5p, was the highest increased circulating miRNA but other species were elevated to comparable degrees (miR-885-5p, miR-151-3p) or were ranked higher by random forest analysis in terms of ability to report injury (miR-382-5p). Circulating miRNAs are stable because they are either bound to proteins or encapsulated in extra-cellular vesicles (or both). The presence of miRNA in vesicles such as exosomes is controversial, with some studies reporting very low amounts in the exosomal cargo^21^. Also extra-cellular vesicles contain Ago2 suggesting there is not a clear division between the two circulating fractions. For the first time we demonstrate that human miR-122-5p circulates bound to the protein Ago2 and this fraction increases with liver injury. Ago2-bound miR-885-5p also increases with injury and both these miRNAs were localised to hepatocytes by *in situ* hybridization on human liver explants removed at transplantation for acetaminophen toxicity. These data suggest release of miR-122 and miR-885 from the same cells attached to the same carrier protein.

The 3 highest fold increase miRNAs and the leading discriminator in our model were tested for specificity. Across all our human array studies and in mice models miR-382-5p decreased in circulating concentration with liver injury. The mechanism of this circulating decrease of miR-382-5p is unknown, but conceptually could be either a decrease in production (such as reduced gene transcription in the injured liver) or increased clearance. Given that more miRNAs increase than decrease in the circulation with acute liver injury it would require a highly selective process of altered clearance to explain the specific decrease in miR-382-5p and decreased tissue expression seems more plausible. miR-122-5p, miR-885-5p, miR-151-3p and miR-382-5p reported acute liver injury due to causes other than acetaminophen, which is consistent with them being liver specific and demonstrates that this panel has utility in the diagnosis of acute liver injury due to multiple causes. Furthermore, none of this panel changed with kidney injury in humans or mice, which also supports liver specificity and validates their use in patients with liver and kidney injury, which commonly co-exist in life-threatening acute liver failure. There were miRNA species that changed with kidney dysfunction and they consistently decreased in circulating concentration. A general fall in circulating miRNA concentrations has been reported in chronic kidney disease and suggested to be due increased blood RNAase activity^22^. Our data would suggest AKI also reduces most, but not all, circulating miRNAs. In the present study the performance of the leading miRNA, miR-19a, was modest with regard to separating liver injury from liver and kidney injury (ROC-AUC 0.76). However, human diagnostic performance may be improved when multiple time points post-drug exposure are analysed in larger studies. This is supported by the case reported in this paper, which demonstrated a decrease in miR-19a before serum creatinine was elevated. By definition, the patients with kidney dysfunction had more severe acetaminophen toxicity and differences in circulating miRNAs could reflect more severe liver injury rather than kidney injury *per se.* Whether miRNA species such as miR-19a are truly kidney specific or represent liver toxicity prognosis markers is an important area for further development.

It is widely reported that miRNAs translate across species; for example, we have reported that miR-122-5p reports liver injury in cell models, zebrafish, rodents and humans. Our array data are consistent with smaller acetaminophen studies performed in rodents: 586 miRNA species were measured in mice^14^ and 750 miRNA species were measured in rats^23^. In addition to miR-122-5p, the miRNA species miR-22, miR-29b, miR-29c, miR-130a and miR-193 were increased in both mice and humans. In rats, in addition to miR-122-5p, the increase of miR-22, miR-193 and miR-194 was in accordance with our human data^23^. This demonstrates the translational potential of miRNAs across rodents and humans. However, the data presented in this study clearly demonstrate that miR-885-5p does not increase with acetaminophen toxicity in mice, an important limitation. Comparative biomarker profiles for miR-122-5p, miR-885-5p, miR-151-3p and miR-382-5p are summarized in supplementary Table 5.

Although miR-122-5p had the highest fold increase in APAP-TOX patients, it was ranked 11th place in the miRNA panel, suggesting that other microRNA species may have greater clinical utility. As a first step in determining the clinical utility of our miRNA panel, the performance of selected members was compared with ALT with regard to patient stratification at first presentation to the ED. These patients were recruited from a different hospital to the training and test patient sets (London as compared to Edinburgh). ROC analysis revealed that miR-122-5p was superior to ALT activity with regard to predicting APAP-TOX in early APAP patients. This observation confirms our previous investigation, in which miR-122-5p displayed superior predictive value over ALT activity at the hospital ‘front door’, in a separate cohort of patients from a different hospital^16^. miRNA species were identified which were stable across patients with and without organ injury. These internal normalizers can be used to report sample degradation and failed miRNA extraction from plasma. Therefore, they are of value for future studies of acute liver injury providing their stability is confirmed in each specific clinical study. When normalized by any of the stable miRNAs, miR-122-5p was superior to ALT. At first presentation, miR-122-5p was also superior to other miRNAs with regard to prediction of subsequent liver injury. This important result may reflect the high abundance of miR-122-5p within the hepatocyte compared to other miRNAs, its high degree of tissue selectivity, potential different pathways of release from injured cells (although miR-885-5p is also bound to same protein), different stability in the circulation or miR-122-5p having a higher baseline circulating concentration. Interestingly, combining miR-122-5p with miR-483-3p resulted in an increase in predictive accuracy (as judged by the largest area under the ROC curve). While this is hypothesis generating, it may be that combining miRNAs that increase and decrease represents the optimal biomarker qualification strategy.

In conclusion, miRNA panels are associated with liver and kidney toxicity in patients with acetaminophen overdose. These panels include novel miRNAs not previously described as toxicology biomarkers. For patient stratification at first presentation to hospital miR-122-5p is the lead miRNA candidate for clinical development, possibly in combination with miR-483-3p. Use of miRNA biomarkers could allow earlier prediction of patients at risk of organ injury and enable targeted intervention in these patients; furthermore they could allow earlier prediction of patients at low risk and enable shorter antidotal treatment regimens and reduce length of hospital stay.

## Patients and Methods

### Patient Cohorts

The local research ethics committee approved the study and informed consent was obtained from all patients before entry into the study. The study was carried out in accordance with the approved relevant guidelines.

### Acetaminophen toxicity (APAP-TOX) patients

A total of 68 adult patients (aged 16 years and above) admitted to the Royal Infirmary of Edinburgh (RIE), UK with ALI, secondary to acetaminophen ingestion, were entered into the study. ALI was defined as a sudden deterioration in liver function in the absence of a history of chronic liver disease with a clear history of excess acetaminophen ingestion. All patients received intravenous NAC treatment.

There were two separate cohorts within this APAP-TOX group. First, patients admitted to the Clinical Toxicology Unit at RIE with a peak serum ALT activity greater than 3 x upper limit of normal (>150 U/L) formed the training set (N = 27). Second, patients admitted to the Scottish Liver Transplantation Unit at RIE with peak ALT > 150 U/L formed the test set (N = 41). There was no patient overlap between training and test sets. Blood samples with peak hospital stay ALT activity were analysed in both training and test sets.

### Acetaminophen no toxicity (APAP-no TOX) patients

67 patients were recruited from RIE. The entry criterion was a history of acetaminophen ingestion in overdose that required treatment with NAC as per UK guidelines at the time of hospital admission^24^. All patients had a plasma acetaminophen concentration above the Prescott nomogram, which confirmed potentially toxic exposure. Blood was collected at the end of the intravenous NAC infusion for measurement of serum ALT activity and absence of liver injury was confirmed by a normal serum ALT activity (<50 U/L). APAP-no TOX patients were age- and sex-matched with the training and test set APAP-TOX patients. There was no patient overlap between training and test set. Blood from the end of NAC treatment was used in this study.

### Acute liver injury not due to acetaminophen (Non-APAP ALI)

Samples from 5 subjects with ALI not due to acetaminophen were recruited to this study. The causes of injury were autoimmune hepatitis, primary graft non-function, small-for-size syndrome, malignancy and clarithromycin-induced liver injury.

### First presentation to hospital patients (APAP-early)

These patients (total N = 67) were all recruited from St Thomas’ Hospital, London UK. Inclusion criteria were: adult with a clear history of excess acetaminophen ingestion and a timed blood acetaminophen concentration that was judged by the treating physician to necessitate hospital admission for intravenous NAC therapy, as per UK guidelines at the time of study. All patients completed the full course of NAC treatment. For all study participants, demographic data and blood results were recorded. ALI was defined as peak serum ALT activity greater than 3x the upper limit of the normal range (>150 IU/L), the UK indication for continuing NAC therapy after completion of the initial regimen. The first blood sample at presentation to hospital was collected for the APAP-early cohort.

In all groups, blood samples were centrifuged at 1000 × g for 15 minutes at 4 °C. The supernatant was then separated into aliquots and stored at −80 °C until analysis.

### MicroRNA profiling of training set

In the training set 27 APAP-TOX samples were compared with 27 APAP-no TOX samples. The methods for profiling are described in the supplemental methods.

### MicroRNA profiling of test set

In the test set 41 APAP-TOX samples were compared with 40 APAP-no TOX samples. miRNA species were selected based on fold change from the training set results. Sample processing was as described for the training set and real-time PCR was again performed on a Fluidigm BioMark HD.

### Ago2-associated miRNA isolation

Magna Bind goat anti-mouse IgG magnetic bead slurry, 100 μL, (Thermo Scientific, Waltham, USA) was incubated with 10 μg of mouse monoclonal anti-Ago2 (Abcam, Cambridge, UK) or mouse normal IgG (Santa Cruz Biotechnology, Dallas, US) antibodies for 2 h at 4 °C. The antibody-coated beads were then added to plasma and incubated overnight at 4 °C with rotation. Beads were washed and each sample then eluted in RNAse free water before QIAzol was added for RNA isolation. Ago2 isolation was determined by western blot analysis.

### Targeted miRNA measurement

Selected miRNAs were quantified in plasma samples by RT-qPCR as described in the supplemental methods. *In situ* hybridisation was performed on human liver removed at the time of transplantation (explant) using methods described in our previous study^13^. The patients had severe acute liver failure secondary to acetaminophen overdose.

### Mouse models of liver and kidney toxicity

Mouse studies were in accordance the Animals (Scientific Procedures) Act 1986 and approved by the local Animal Ethics Committee. In brief, male CD1 mice (28–33 g) were treated with either 0.9% saline, cisplatin (20 mg/kg) or acetaminophen (350 mg/kg) IP. At 24–72 hours after treatment, blood was collected via cardiac puncture and allowed to clot before isolation of serum which was stored at −80 °C. Tissue samples were collected and either snap frozen or fixed in neutral buffered formalin (10%).

### Statistical analysis

Data are presented as median and range or interquartile range (IQR). Each dataset was analysed for normality using a Shapiro-Wilk test. For nonparametric datasets, comparisons were made using the Mann-Whitney U test or Wilcoxon matched-pairs signed rank test. All calculations and receiver operator characteristic (ROC) curves were performed using GraphPad Prism software (GraphPad Software, La Jolla, CA). Profiling data were analysed by random forests to give an estimate of how well we can classify individuals in a new data set into each group. Random forests create a set of classification trees based on continual sampling of the experimental units and compounds. Each observation is then classified based on the majority votes from all of the classification trees. Details of random forest analysis can be found in the supplemental methods.

## Additional Information

**How to cite this article**: Vliegenthart, A. D. B. *et al.* Comprehensive microRNA profiling in acetaminophen toxicity identifies novel circulating biomarkers for human liver and kidney injury. *Sci. Rep.* **5**, 15501; doi: 10.1038/srep15501 (2015).

## Supplementary Material

Supplementary Methods and Results

Supplementary Dataset 1

## Figures and Tables

**Table 1:** Comparison of circulating miRNAs in acetaminophen-induced acute liver injury patients with and without kidney dysfunction.

**miRNA**	**Fold change**	***P*** **value**	**ROC-AUC (95% CI)**	***P*** **value**	**SENS (95% CI)**
miR-19a-3p	−3.76	**<0.0001**	0.76 (0.66–0.86)	**<0.0001**	0.51 (0.35–0.67)
miR-19b-3p	−4.10	**0.001**	0.73 (0.61–0.85)	**0.001**	0.27 (0.12–0.46)
miR-192-5p	−1.66	**0.001**	0.73 (0.61–0.85)	**0.001**	0.20 (0.08–0.39)
miR-34a-5p	−5.71	**0.003**	0.71 (0.58–0.83)	**0.003**	0.20 (0.08–0.39)
miR-3187-3p	−5.30	**0.005**	0.70 (0.58–0.83)	**0.005**	0.47 (0.28–0.66)
miR-122-5p	1.28	0.92	0.51 (0.36–0.65)	0.92	0.27 (0.12–0.46)
miR-885-5p	1.25	0.73	0.53 (0.38–0.67)	0.72	0.33 (0.17–0.53)
miR-151a-3p	1.28	0.27	0.58 (0.44–0.72)	0.27	0.10 (0.02–0.27)
miR-382-5p	1.46	0.67	0.53 (0.39–0.68)	0.67	0.17 (0.06–0.35)

ROC-AUC (area under the curve with 95% CI), sensitivity (SENS with 95% CI) at 90% specificity and statistical significance are given. Negative fold change means lower with kidney dysfunction.
