# Supplementary material for: Comprehensive microRNA profiling in acetaminophen toxicity identifies novel circulating biomarkers for human liver and kidney injury
Source: Sci Rep. 2015 Oct 22;5:15501. doi: 10.1038/srep15501 (PMC4614545; doi:10.1038/srep15501)
Supplement: Supplementary Methods and Results [file srep15501-s1.pdf]

Comprehensive microRNA profiling in acetaminophen toxicity identifies novel circulating biomarkers for human liver and kidney injury

A D B Vliegenthart, J M Shaffer, J I Clarke, L E J Peeters, A Caporali, D N Bateman, D M Wood, P I Dargan, D G Craig, J K Moore, A I Thompson, N C Henderson, D J Webb, J Sharkey, D J Antoine, B K Park, M A Bailey, E Lader, K J Simpson, J W Dear

**Supplementary Tables**	Page 2-6
**Supplementary Figure**	Page 7-8
**Supplementary Methods**	Page 9-13

Supplementary Tables

Supplementary Table 1. Patient demographics and clinical chemistry results in the training and test set.

	**Training set**		**Test set**	
	**APAP-TOX**	**APAP-no TOX**	**APAP-TOX**	**APAP-no TOX**
Number	27	27	41	40
Sex (male:female)	10:17	11:16	13:28	15:25
Age years (IQR)	39 (26-54)	38 (25-55)	39 (31-49)	38 (29-49)
ALT IU/l (IQR)	2150 (487-4444)	16 (13-26)	3661 (2454-6306)	20 (12-25)
INR (IQR)	1.5 (1.3-2.1)	1.1 (1.0-1.2)	3.4 (2.3-6.2)	1.1 (1.0-1.1)
Creatinine µmol/L (IQR)	66 (58-170)	62 (55-80)	157 (66-230)	64 (56-69)
Creatinine >110 µmol/L (number)	8	0	22	0

ALT, alanine aminotransferase; INR, International Normalized Ratio. Data are presented as median values.

Supplementary Table 2. Patient demographics and clinical chemistry results in patients with acetaminophen induced liver injury with normal renal function (APAP-ALI no AKI) and with abnormal renal function (APAP-ALI with AKI)

	APAP-ALI no AKI	APAP-ALI with AKI
Number	38	30
Sex (male:female)	(14:24)	(14:16)
Age years (IQR)	40 (27-58)	41 (31-49)
ALT IU/l (IQR)	2307 (651-3455)	4083 (2920-7398)
INR (IQR)	1.9 (1.4-2.8)	3.4 (2.1-6.1)
Creatinine µmol/L (IQR)	61 (56-73)	209 (120-286)

ALT, alanine aminotransferase (ALT); INR, International Normalized Ratio. Data are presented as median values. *** $P < 0.0001$; ** $P < 0.001$ (Mann Whitney test).

Supplementary Table 3. Normfinder assessment of 50 most stable expressed microRNA, including stability value.

microRNA	Stability value
hsa-miR-1287	0.000012
hsa-miR-4289	0.000013
hsa-miR-1913	0.000015
hsa-miR-5194	0.000016
hsa-miR-671-3p	0.000017
hsa-miR-1260a	0.000018
hsa-miR-324-3p	0.000018
hsa-miR-423-5p	0.000018
hsa-miR-572	0.000019
hsa-miR-195-5p	0.000019
hsa-miR-718	0.000019
hsa-miR-23b-3p	0.000020
hsa-miR-505-3p	0.000020
hsa-miR-151a-5p	0.000020
hsa-miR-21-5p	0.000020
hsa-miR-874	0.000021
hsa-let-7b-5p	0.000021
hsa-miR-375	0.000021
hsa-miR-564	0.000021
hsa-miR-101-3p	0.000021
hsa-miR-766-3p	0.000021
hsa-miR-421	0.000022
hsa-miR-30e-5p	0.000022
hsa-miR-433	0.000022
hsa-miR-30d-5p	0.000022
hsa-miR-30a-5p	0.000022
hsa-miR-4291	0.000022
hsa-let-7d-3p	0.000022
hsa-miR-193b-3p	0.000023
hsa-miR-19a-3p	0.000023
hsa-let-7b-3p	0.000023
hsa-miR-4286	0.000023
hsa-miR-15b-3p	0.000024
hsa-miR-1307-3p	0.000024
hsa-miR-3663-3p	0.000027
hsa-miR-92a-3p	0.000028
hsa-miR-3689a-5p // hsa-miR-3689b-5p // hsa-miR-3689e	0.000033
hsa-miR-1260b	0.000038
hsa-miR-29a-3p	0.000042
hsa-miR-19b-3p	0.000042
hsa-miR-181b-5p	0.000047
hsa-let-7c	0.000049
hsa-miR-3187-3p	0.000050
hsa-miR-29b-1-5p	0.000055
hsa-miR-25-3p	0.000069
hsa-miR-4695-5p	0.000073
hsa-miR-484	0.000076
hsa-miR-486-5p	0.000116
hsa-miR-1238-3p	0.000166
hsa-miR-3183	0.000169

Supplementary Table 4. Patient demographics of the early acetaminophen overdose patient cohort.

Number	67
Sex (male:female)	34:33
Age years	39 (26-49)
Amount of acetaminophen ingested g	16 (12-30)
Time from ingestion to first blood sample hr	6 (4-16)
Admission acetaminophen concentration mg/L	101 (24-139)
Admission serum creatinine μmol/L	66 (56-77)
Admission billirubin μmol/L	6 (4-11)
Admission ALT activity IU/L	20 (14-34)
Admission ALP activity IU/L	72 (56-89)
Admission GGT activity IU/L	31 (20-73)
Admission INR	1.0 (1.0-1.1)
Number with admission ALT<ULN	58
Number with peak ALT > 3x ULN	13
Number with peak ALT > 1000 IU/L	5
Number with admission INR > 1.5	1
Number with peak INR > 1.5	6

ALT, alanine aminotransferase; ALP, alkaline phosphatase; GGT, gamma glutamyl transpeptidase; INR, International Normalized Ratio; ULN, upper limit of normal. Data are presented as median values with IQR.

Supplementary Table 5. miRNA biomarker profiles

miRNA species	Change with APAP	Reports non-APAP ALI	Reports APAP-ALI in mice	Change with kidney injury	Ago2 bound	More sensitive than ALT for early injury
miR-122-5p	Increase	Yes	Yes	No	Yes	Yes
miR-885-5p	Increase	Yes	No	No	Yes	No
miR-151a-3p	Increase	Yes	Yes	No	No	No
miR-382-5p	Decrease	Yes	Yes	No	Yes	No

Supplementary Figures

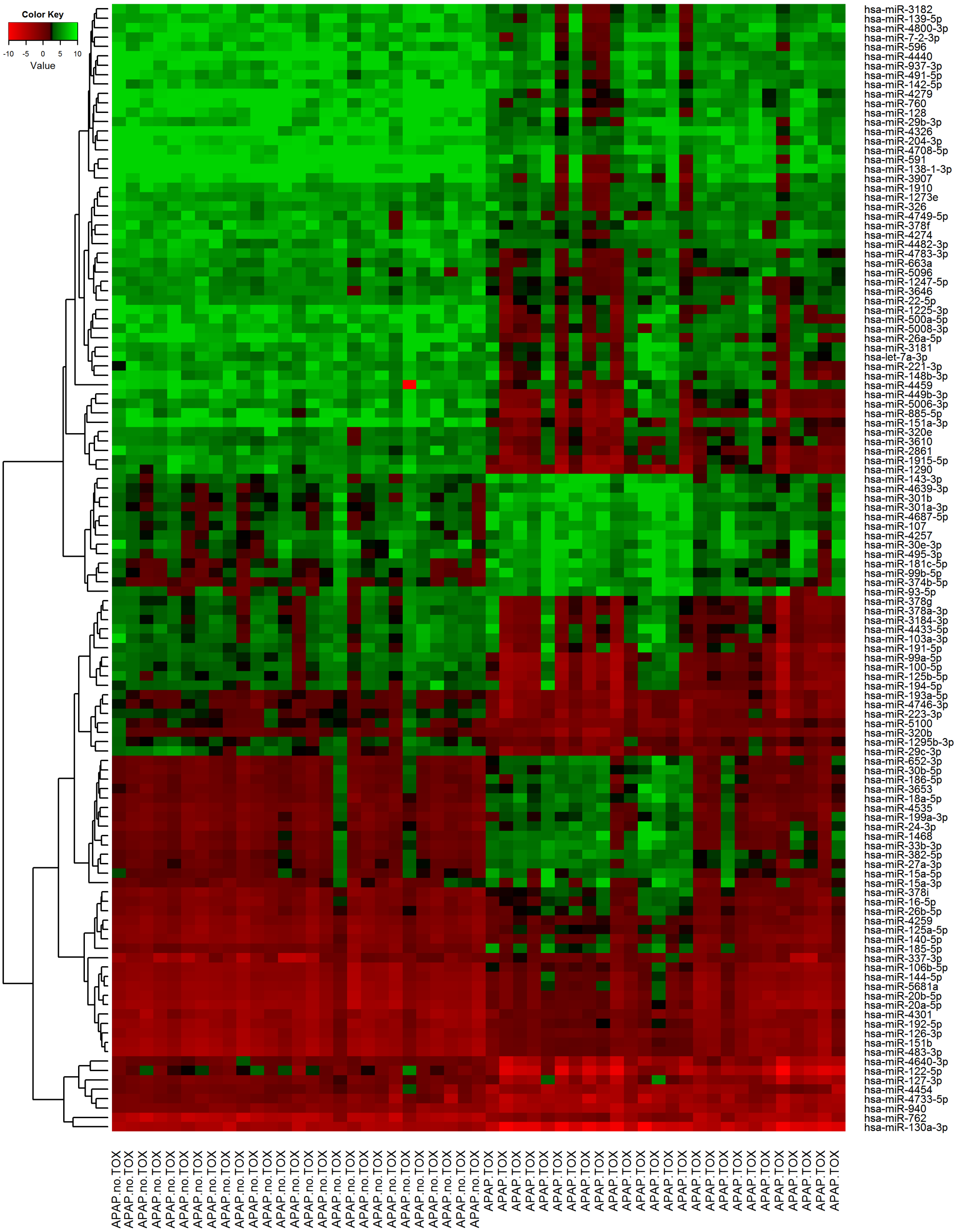

Supplementary Fig. 1. Heatmap displaying cluster analysis of >3-fold increased/decreased circulating miRNAs in acetaminophen no toxicity (APAP-no TOX) and acetaminophen toxicity (APAP-TOX) patients from the training set. Each row represents a miRNA, and each column represents a patient sample.

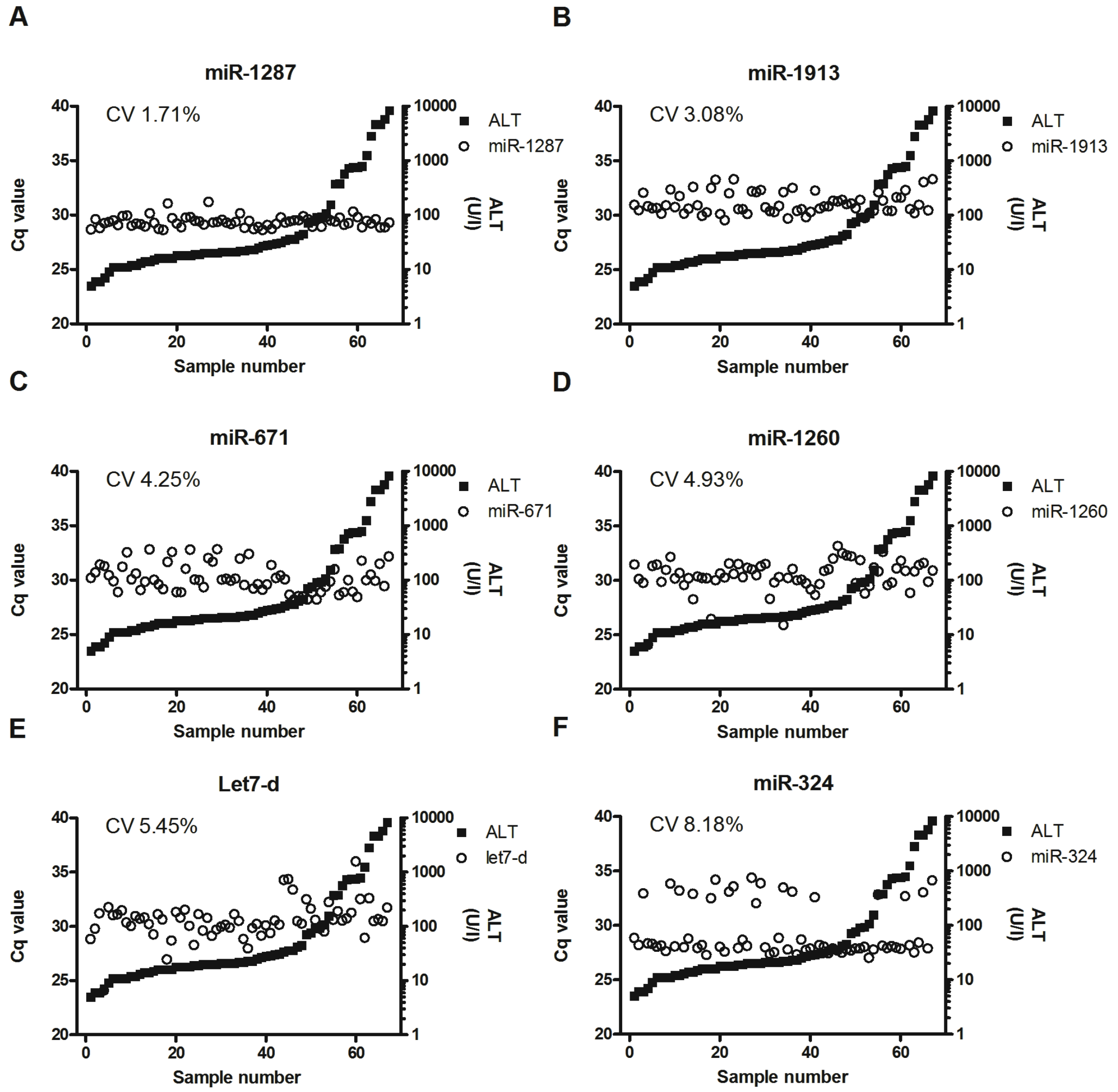

Supplemental Fig 2. Graphical representations of the Ct values of 6 endogenous normalizers and ALT. Samples are from the APAP-early cohort. The coefficient of variation (CV) is recorded on each graph.

Supplementary methods

MicroRNA profiling

miRNA PCR RNA isolation and Sample Quality Control

RNA was isolated from 200 µl plasma samples using the miRNeasy Serum/Plasma Kit (Qiagen, Venlo, Netherlands). RNA was eluted in a fixed volume of 14 µl and stored at -80^{0}C. Samples were evaluated for suitability by using the miScript PCR System (Qiagen, Venlo, Netherlands). Briefly, 1.5 µl of each eluate was reverse transcribed into cDNA using the miScript II RT Kit with miScript HiSpec Buffer. The reactions were incubated for 60 minutes at 37°C followed by a heat inactivation step for 5 minute at 95°C. One µl of each 10 µl cDNA synthesis was then diluted eleven-fold and assessed for a) the presence of common miRNA and for the absence of RT-PCR inhibitors using the miScript SYBR Green PCR Kit and the miScript miRNA QC PCR Array. Real-time PCR was performed on an ABI-7900HT (Applied Biosystems, Foster City, CA) using the recommended miScript cycling parameters.

Phase I: Determine Expressed miRNAs

A collection of 18 randomly chosen training set APAP no TOX samples and 18 randomly chosen training set APAP-TOX samples were combined into two control pools and two liver injury sample pools, respectively, with each pool containing RNA from 9 samples. cDNA synthesis and real-time PCR were performed using the miScript PCR System. Briefly, 7.5 µl of total RNA eluate from each of the four pooled samples was reverse transcribed into cDNA using the miScript II RT Kit with miScript HiSpec Buffer. Each cDNA synthesis was diluted

to a final volume of 550 µl using RNase-free water. For real-time PCR analysis of miRNA expression, diluted cDNA (1 µl per 4 wells) was analyzed using the miScript SYBR Green PCR Kit and the Human miRNome miScript miRNA PCR Array (miRBase V18, 1809 miScript Primer Assays). Real-time PCR was performed on an ABI-7900HT (Applied Biosystems, Foster City, CA) using the miScript cycling program. A miRNA was deemed to be expressed if its C_t value was less than 35 with a single, sharp melt peak in at least one of the four pools. A total of 356 miScript Primer Assays (or roughly 20% of the miRNome) were selected for Phase II screening.

Phase II: Determine Differentially Expressed miRNAs

cDNA synthesis, cDNA preamplification, and real-time PCR were performed as described earlier for each of the 54 samples. Each cDNA synthesis was diluted 5-fold and one-tenth was preamplified using the miScript Microfluidics PreAMP Kit in combination with a Custom miScript PreAMP Primer Mix containing the 356 miScript Primer Assays identified as 'expressed' in Phase I. Twelve cycles of preamplification was performed using the manufacturer recommended 384-plex preamplification protocol. Amplified cDNA was diluted to a final volume of 125 µl using RNase-free water and assessed in real-time PCR using the miScript Microfluidics PCR Kit and a Custom miScript miRNA PCR Array containing the 356 expressed assays from Phase I. Real-time PCR was performed on a Fluidigm BioMark HD (Fluidigm, San Francisco, US) using the miScript cycling program for the Fluidigm BioMark, which consists of an initial thermal mix stage (50°C for 2 minutes, 70°C for 30 minutes, and 25°C for 10 minutes) followed by a hot start at 95°C for 15 minutes and 40 cycles of 94°C for 15 seconds, 55°C for 30 seconds, and 70°C for 30 seconds. For data processing, C_t values were calibrated for RNA recovery using the cel-miR-39-3p assay

(which detects a synthetic miRNA spiked in to each sample during sample prep), and any miRNA assay with a $C_t > 23$ was deemed to be not expressed. For data analysis, the $\Delta\Delta C_t$ method of relative quantification was used. The C_t values used for calculating the fold changes were normalized using the Global C_t mean. The miRNAs included in the C_t mean were expressed in all samples at a $C_t < 23$. The C_t values used for the random forest analysis were normalized using invariant miRNAs.

Random forest analysis of profiling test set data

Classifier training was performed using APAP-no TOX (27 samples) versus APAP-TOX (26 samples). The random forest classification method was used for two independent steps: (1) selection of the most predictive 16 miRNAs from the full set of experimentally selected 92 miRNAs and 1 snoRNA, and (2) training a final 16-miRNA classification model.

Step 1: selection of 16 miRNAs. To select 16 miRNAs from the 93 measured without feature selection bias, we drew 250 bootstrap subsamples of the full training set, without replacement. The subsamples were chosen to contain about 80% of the smaller TOX class and an equal number of no-TOX samples. This gave 20 APAP-noTOX versus 20 APAP-TOX. For each subsample, random forest classifier was trained, saved by the gene importance rank (by sorting according to the marginal decrease in out-of-bag prediction accuracy when the gene's expression measurements are scrambled), saved by predictions on the left-out samples and then the random forest model was discarded. After all 250 iterations were complete, 93 genes were sorted by their median importance rank across all 250 bootstrap trainings, and the top 16 genes were selected. In addition to using the 250 bootstrap

subsamples to obtain the 16 potentially most predictive genes, we also used the samples left out of training in each subsample to assess class separation magnitude and model error.

Step 2: final classifier training. Using the final 16 genes only, we trained a new random forest classifier using all training set samples, and saved this for use in class prediction on new samples. The performance of this final 16 gene classifier was tested using our independent test set samples. The classifier gives the probability that each sample is APAP-TOX. This classification was performed blind to the true sample grouping.

MicroRNA measurement by PCR

miRNA timecourse, miRNA in Ago2 fraction and miRNA in mice

RNA was isolated from 50 µl plasma samples using the miRNeasy Serum/Plasma Kit (Qiagen, Venlo, Netherlands). RNA was eluted in a fixed volume of 14 µl, after which 2 µl was reverse transcribed using the TaqMan MicroRNA Reverse Transcription Kit using stem-loop primers (Applied Biosystems, Foster City, CA) for each target miRNA species following the manufacturer's instructions. Then, 1.33 µL of cDNA was used in the PCR mixture using the specific PCR probes (Applied Biosystems, Foster City, CA). Levels of miRNAs were measured using the Light Cycler 480 (Roche, Basel, Switzerland).

miRNA measurement - APAP - early

RNA was isolated from 50 µl plasma samples using the miRNeasy Serum/Plasma Kit (Qiagen, Venlo, Netherlands). RNA was eluted in a fixed volume of 14 µl, after which 5 µl of each eluate was reverse transcribed into cDNA using the miScript II RT Kit (Qiagen, Venlo, Netherlands) following manufacturers instructions. The synthesized cDNA was ten-fold

diluted and used for cDNA template in combination with the miScript SYBR Green PCR Kit (Qiagen, Venlo, Netherlands) using the specific miScript assays (Qiagen, Venlo, Netherlands). Real-time PCR was performed on a Light Cycler 480 (Roche, Basel, Switzerland) using the recommended miScript cycling parameters. Ct values greater than 35 were considered to be negative for that miRNA.
